# Supplementary material for: Evolution of sex differences in cooperation can be explained by trade-offs with dispersal
Source: PLoS Biol. 2024 Oct 24;22(10):e3002859. doi: 10.1371/journal.pbio.3002859 (PMC11500963; doi:10.1371/journal.pbio.3002859)
Supplement: S1 File — Text B. Additional Encounternet methods. Text C. Impact of unknown dispersal events on age-specific dispersal probabilities of natal subordinates. Text D. Sex difference in the strategy used to acquire dominance via dispersal. (DOCX) [file pbio.3002859.s001.docx]

##

**S1 File for:**

**Evolution of sex differences in cooperation can be explained by trade-offs with dispersal**

Pablo Capilla-Lasheras^1, 2, 3, *^, Nina Bircher^4^, Antony M. Brown^1^, Xavier Harrison^1^, Thomas Reed^5^, Jennifer E. York^6,7^, Dominic L. Cram^6^, Christian Rutz^8^, Lindsay Walker^1^, Marc Naguib^4^, Andrew J. Young^1, *^

* pacapilla@gmail.com (PC-L); A.J.Young@exeter.ac.uk (AJY)

^1^ Centre for Ecology and Conservation, University of Exeter, Penryn, UK

^2^ Bird Migration Unit, Swiss Ornithological Institute, Sempach, Switzerland

^3^ School of Biodiversity, One Health and Veterinary Medicine, University of Glasgow, Glasgow, Scotland

^4^ Behavioural Ecology Group, Wageningen University & Research, Wageningen, The Netherlands

^5^ School of Biological, Earth and Environmental Sciences, University College Cork, Cork, Ireland

^6^ Department of Zoology, Downing Street, University of Cambridge, Cambridge, UK.

^7^ Department of Zoology and Entomology, University of Pretoria, Pretoria, Republic of South Africa

^8^ Centre for Biological Diversity, School of Biology, University of St Andrews, Sir Harold Mitchell Building, St Andrews, UK

**S1 File includes:**

[**A - Quantifying the contributions of natal subordinates to cooperative provisioning** 3](#_Toc178584374)

[**B - Additional Encounternet methods** 4](#_Toc178584375)

[**C - Impact of unknown dispersal events on age-specific dispersal probabilities of natal subordinates** 5](#_Toc178584376)

[**D - Sex difference in the strategy used to acquire dominance via dispersal** 6](#_Toc178584377)

[**References in S1 File** 7](#_Toc178584378)

# **A - Quantifying the contributions of natal subordinates to cooperative provisioning**

To determine the rates at which helpers within their natal groups (i.e., all subordinate, and therefore non-breeding, group members still residing within the group in which they born) cooperatively provision the nestlings of the dominant pair, provisioning behaviour was recorded for all breeding attempts throughout the study period (2007 – 2016) in which the nestlings survived to the standard recording days. A video camera was placed on a low tripod below the nest to record the birds entering the nest on the mornings of the 6^th^ to 12^th^ days (inclusive) after the first egg in the focal clutch hatched (days that cover the period of highest nestling demand; nestlings fledge from the 17^th^ day onwards). At least 5 days before video recording started, we caught every bird in the group apart from the dominant female (to avoid the risk of triggering abandonment) to confirm group composition and mark each bird’s vent feathers with a uniquely positioned black dye mark to facilitate their identification on provisioning videos. On recording days, video cameras were set up at a standardized time following sunrise to record provisioning for approximately 3 hours per nest per day (yielding a total of 289 days of recording over the 177 monitored broods in groups with one or more helpers within their natal group; median of 2, range 1-6, days per brood). Within-nest cameras have confirmed that all nest visits in which the birds were not conspicuously carrying grass (events that were readily excluded from our provisioning rate calculations below), entail the visiting bird carrying a single food item to the nest and delivering it to the chicks [1]. The videos were subsequently transcribed to determine the timing of each provisioning visit, the duration of the visit (from the bird entering the nest to exiting), and the identity of the provisioning bird (using its unique vent mark and information from its leg rings and sex-specific bill colouration). Whenever the bill of the provisioning bird was visible on their approach to the nest, we also categorised the size of the food item being delivered; simply ‘large’ if it was evidently longer than the bird’s bill or ‘short’ if it was not.

We used the resulting provisioning data set to calculate the number of provisioning visits that each group member made during each observation period (termed ‘provisioning number’ within our methods). In some cases, we were unable to reliably identify all group members, adding uncertainty to our provisioning number estimates. We therefore excluded any provisioning number estimates derived from observation periods in which there were five or more provisioning visits in which the feeder was of uncertain identity (72 provisioning number estimates excluded). To address the residual uncertainty in the provisioning number estimates derived from the observation periods containing one to four feeds by birds of uncertain identity, we set each bird’s provisioning number estimate for these observation periods to be the integer midway between the lowest and highest possible provisioning number for that bird in that period given any partial identity information that was available for the feeders of uncertain identity. This yielded a final dataset containing 1,338 daily provisioning number measures for subordinates (helpers) of known sex within their natal groups for whom age was known with less than 180 days of uncertainty.

# **B - Additional Encounternet methods**

To calibrate the base stations, we attached two (transmitter) tags to a wooden pole with one of the tags’ antenna positioned parallel to (vertical), and the other positioned perpendicular to (horizontal), the focal base station’s antenna (to account for different antenna angles when the tags were on free-flying birds [2]. We then held the tags (on the pole) at a fixed distance of two meters from each base station and at a height of 1.70 meters for two minutes, resulting in 48 logged pulses with RSSI values for each base station. We then calculated the mean RSSI value logged by each base station (mean_Basestation_) and the mean RSSI value over all base stations (mean_RSSI_). For the 10 base stations for which mean_Basestation_ was outside the range of mean_RSSI_ ± standard deviation (SD), we adjusted the signal strength (RSSI) value of all logs from these base stations during analysis by adding mean_RSSI_-mean_Basestation_ (to avoid an over or underestimation of the distance between tagged birds and the focal base stations). We did not formally calibrate the individual tags (as well as the base stations), but we did verify qualitatively that all tags were yielding broadly comparable signal strengths at a fixed distance from a given base station prior to deployment. To ensure that the times at which tags were logged at different base stations could be compared over short timescales (to infer tag movements), we regularly checked that the clocks of all base stations were synchronised (yielding a typical deviation of between 0 and 1 seconds; maximum 6 seconds). Base station batteries were changed every 10 days, before they were discharged. Tag signal strength seemed to stay relatively constant during the period of data collection.

We discarded all logs of a given tag from (i) the day on which the tag was deployed and the following day [3] (to allow time for the tagged bird to get used to the tag and recover from any capture-related disturbance), and (ii) a short period following any attempt to catch a member of the tagged bird’s home group (from the time of the capture attempt to the end of the following day; given the potential for capture attempts to impact the birds’ movements). A given tag’s logs were then processed in moving windows of 15 seconds and in time steps of 5 seconds. For each of these 15-second windows, we attempted to assign the tagged bird a ‘best estimate’ location following a conservative set of rules depicted as a flow diagram in S3 Fig and explained in the figure legend. Five birds were assigned to known locations (i.e., locations other than ‘unknown’ in S3 Fig) for a low proportion of their total tracked time (<30%), most likely due to tag failure, and so were excluded from our analyses. The average proportion of time that the remaining 27 tagged birds were assigned known locations was 73%. Note that with the rule set for location assignment (S3 Fig), a tag having weak transmission strength (e.g., due to tag error or signal obstruction) would not cause it to be falsely assigned ‘non-home’ locations (that might later be interpreted as prospecting), because assignment to ‘non-home’ locations required that the tag was logged with a stronger signal strength at a non-home group than the home group (S3 Fig). Consecutive 15 second windows assigned to the same location were combined to represent ‘time periods’ for which our best estimate location was on a given territory (‘unknown’ locations [see S3 Fig legend] were removed from the tag’s location time series prior to this step, as they could occur while the bird was at any location due to the bird’s microenvironment impeding signal transmission).

*A priori* we set the distance threshold to detect extra-territorial forays to > 250m as this approach renders it highly likely that the focal bird itself is > 125m away from the centre of their home territory, because (given the workflow in S3 Fig) its signal strength must have been stronger at a base station receiver > 250m away from home than it was at any other receiver in the array, including the one at the centre of its home territory. As the mean (± SE) distance between the centres of neighbouring territories is 93.7 m (± 4.56 m) in our study population, this minimum plausible distance of 125m from the centre of the tagged bird’s home territory should ensure that ‘forays’ detected using this 250m distance threshold will typically have involved movements beyond the territory-centres of the tagged bird’s neighbouring groups.

# **C - Impact of unknown dispersal events on age-specific dispersal probabilities of natal subordinates**

A small number of unringed adults born outside our study population do enter our study groups each year, suggesting that a small number of adults born within our study population probably also disperse beyond its boundaries each year [4]; unobserved dispersal events that this analysis will therefore miss. As males disperse significantly further than females in this population [4], and accordingly males are also more likely than females to enter our study population from outside [4], it would seem that males are more likely to be making undetected dispersals beyond the boundaries of our study site than females are. The key finding from this analysis, that males show a higher age-specific probabilities of dispersal than females is therefore if anything likely to underestimate the true magnitude of this sex differences in dispersal probability.

# **D - Sex difference in the strategy used to acquire dominance via dispersal**

As subordinate males and females are equally likely to inherit natal dominance (Main text Fig 2), the observed male-bias in age-specific dispersal probability (Main text Fig 1a) appears to reflect a sex difference in the *strategy* used to acquire dominance via dispersal, rather than in the *incidence* of acquiring dominance via dispersal. The data supports this: when subordinates dispersed from their natal groups, males were more likely than females to disperse into non-breeding subordinate positions in other groups (46 of 56 observed natal male dispersals; 19 of 32 observed natal female dispersals; χ^2^ = 4.16, p = 0.041, S10 Table), where they might eventually win dominance or disperse again [4]. Whereas when females dispersed from their natal groups, they were more likely than dispersing males to disperse directly into a dominant position (10 of 56 dispersals for males; 13 of 32 dispersals for females; χ^2^ = 4.16, p = 0.041). Thus, males disperse more frequently than females, but employ a more complex dispersal strategy to acquire dominance often involving staging as a subordinate in other groups prior to dominance acquisition elsewhere, while females disperse less frequently but are more likely to do so directly into dominant positions elsewhere.

# **References in S1 File**

1. Walker L. The evolution and regulation of cooperation in the wild. University of Exeter; 2015.

2. Rutz C, Morrissey MB, Burns ZT, Burt J, Otis B, Clair JJH. Calibrating animal-borne proximity loggers. Methods Ecol Evol. 2015;6: 656–667.

3. Clair JJH, Burns ZT, Bettaney EM, Morrissey MB, Otis B, Ryder TB. Experimental resource pulses influence social-network dynamics and the potential for information flow in tool-using crows. Nat Commun. 2015;6: 7197.

4. Harrison X, York JE, Young AJ. Population genetic structure and direct observations reveal sex-reversed patterns of dispersal in a cooperative bird. Mol Ecol. 2014;23: 5740–5755.
