# Supplementary figures and images for: Evolution of sex differences in cooperation can be explained by trade-offs with dispersal
Source: PLoS Biol. 2024 Oct 24;22(10):e3002859. doi: 10.1371/journal.pbio.3002859 (PMC11500963; doi:10.1371/journal.pbio.3002859)

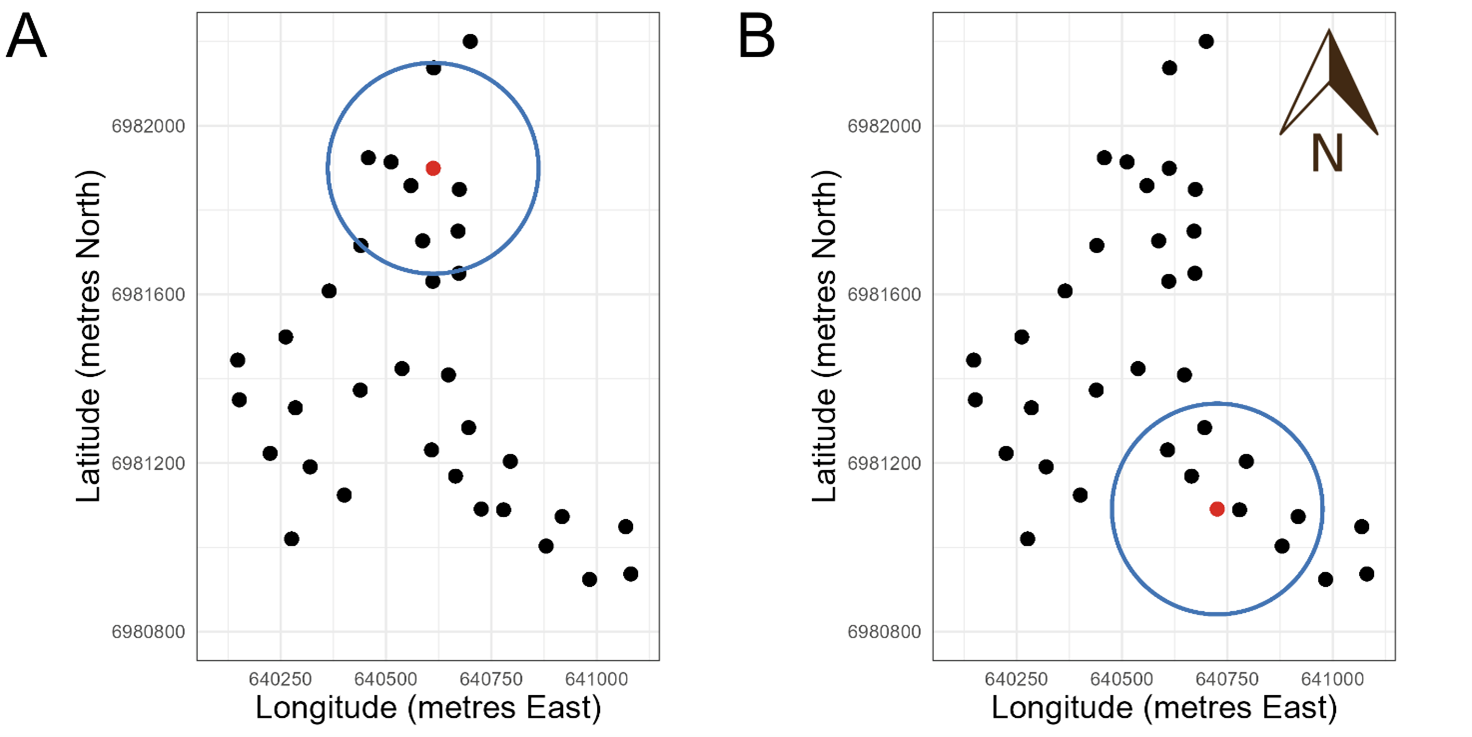

Supplement: S1 Fig — Panel (A) and (B) show the same simplified map of the study site with the locations of the 35 base stations (black and red dots), most of which were placed in the centre of distinct sparrow weaver territories (see Methods). The x and y axes present longitude and latitude (respectively) in metres East and metres North of a given arbitrary location and thus also provide the scale for these maps. In each panel, a circle of 250 meters radius around a focal base station (red dot) is illustrated with a blue line; the only difference between the panels being the location of the focal base station. A single foray was defined as a continuous run (in time) of location estimates which suggested that the bird’s closest base station was >250 m away from the centre of its home territory (i.e., outside blue circle) for at least 15 s. As the mean (± SE) distance between the centres of neighbouring territories was 93.7 m (± 4.56 m), the forays detected with this approach will typically have involved movements beyond the centres of the territories of neighbouring groups. This conservative approach will minimise the chance that a resident bird’s territorial interactions with its neighbouring groups along their shared territory boundary are incorrectly interpreted as extra-territorial prospecting, but is likely to underestimate the true incidence of extra-territorial prospecting by excluding more local forays. The lack of base stations placed within the territories of study groups in the regions outside our core study population will also have left this approach underestimating true foray rate (and likely mean foray distance too). The land to the East and West of the presented array contains no other sparrow weaver territories within the pictured area (and so the focal birds will not have been conducting forays to groups living in those areas). However, there are a small number of widely spaced territories to the North of the array and several also lie close to the array to the South. [file pbio.3002859.s002.png]

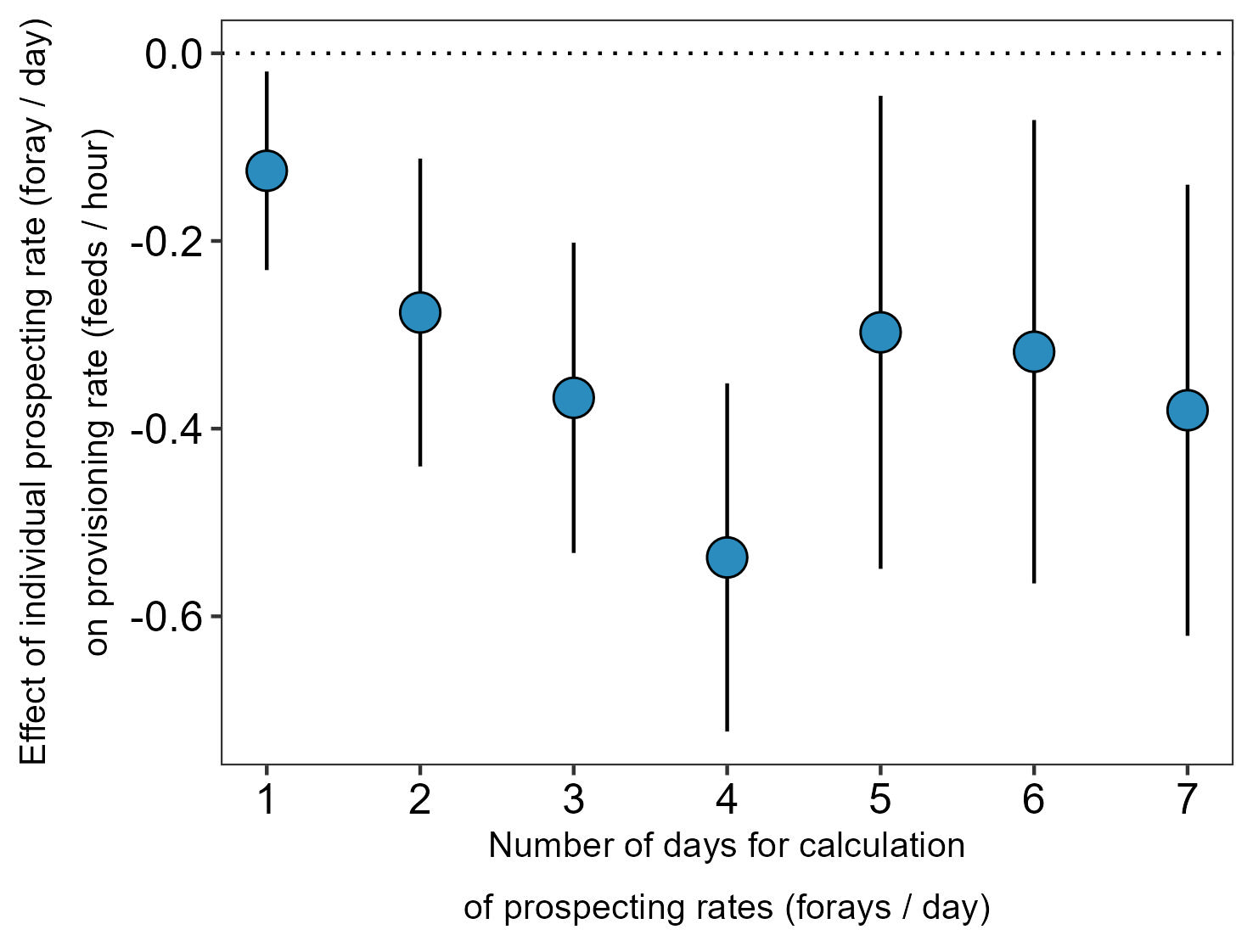

Supplement: S2 Fig — Our analyses within the main paper calculated the prospecting rate over the 3 days prior to the measurement of provisioning rate, as we thought it plausible a priori that any energetic or stress-related costs of prospecting might accumulate and be evident over this timescale. However, on recognising that this decision is somewhat arbitrary we sought to verify, via this sensitivity analysis, that the detected negative covariance between the 2 traits was not particular to this choice of time window. The analysis confirms evidence of negative covariance between the 2 traits over a range of time windows. The initial steady increase in the effect size as the length of the time window considered increases could reflect (i) the timescale over which accumulated costs arising from recent prospecting impact cooperative behaviour, and/or (ii) that, given the modest rate at which prospecting forays occur, the shortest time windows may simply give a poorer-quality estimate of the focal bird’s overall true rate of prospecting. Dots and error bars represent mean model estimates ± SE for the effect of prospecting rate on provisioning rate from the model presented in S9 Table when calculating each bird’s prospecting rate over different time windows. Data and code needed to generate this figure can be found at https://doi.org/10.5281/zenodo.13623047. (PNG) [file pbio.3002859.s003.png]

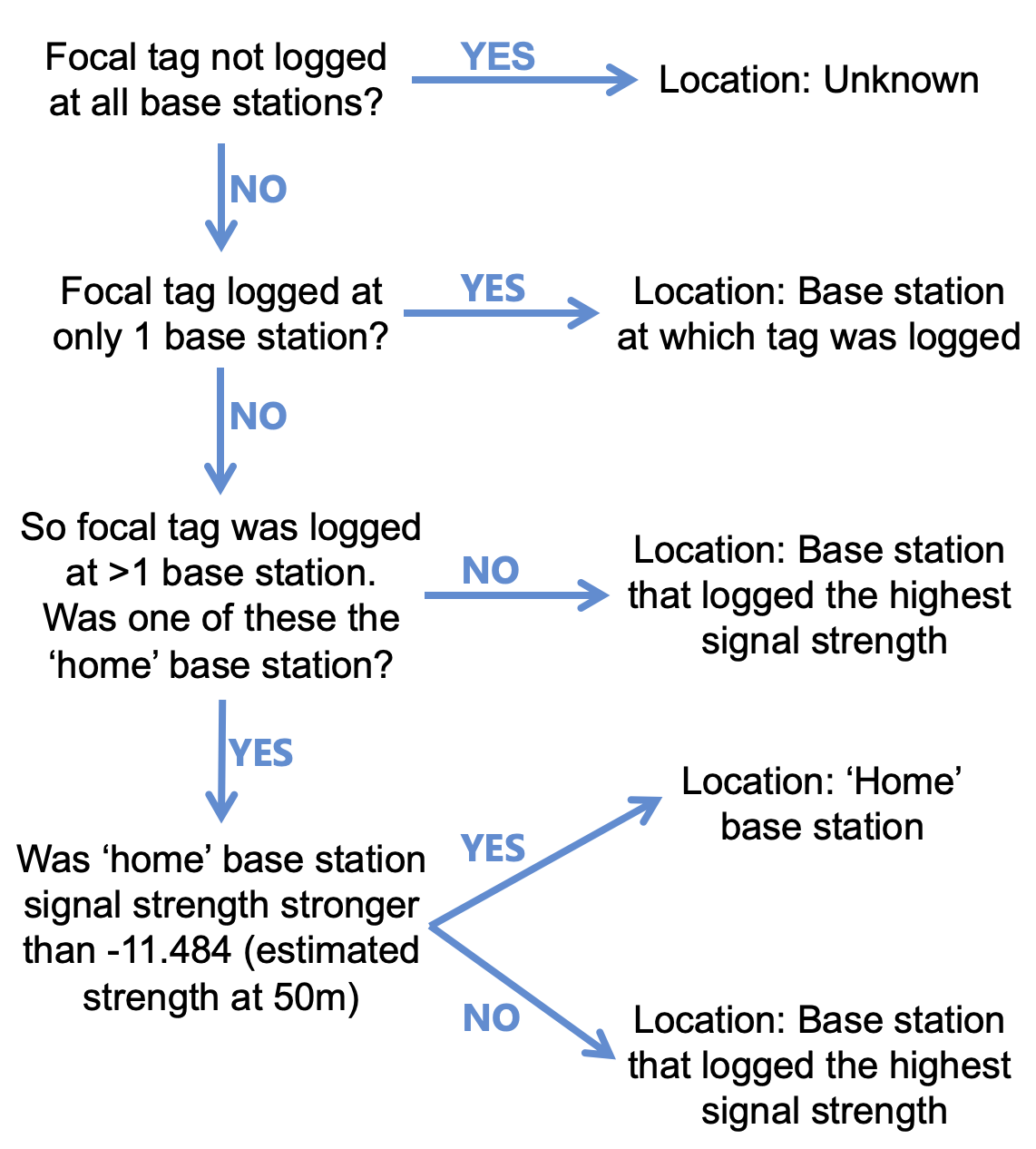

Supplement: S3 Fig — For each 15-s window in the time series of logs for a given bird, we attempted to assign the tagged bird a “best estimate” location via the following set of rules depicted by the flow diagram here. First, if there were no logs at all for the focal tag during the focal time window, we noted the bird’s location as “unknown” (we did not draw spatial inferences from such “unknown” location events as they could reflect the bird being in a microenvironment that impeded signal transmission, such as thick cover). Second, if there were logs for the focal tag from just one base station (indicating that the tag was out of reception range from all other base stations), we assigned the tagged bird the location of the base station at which it was logged. Third, if there were logs for the focal tag from more than one base station but none of them were the tagged bird‘s home base station, we assigned the tagged bird the location of the base station whose logs had the highest mean signal strength. Note that the highest mean signal strength could nevertheless have been weak in this case (e.g., if the bird was well beyond the boundary of our base station array, having prospected out of the study area, its tag might be logged with only a weak signal strength at the base station closest to it on the array periphery; hence us terming these “best estimate” locations). Fourth, if there were logs for the focal tag from more than one base station but one of them was the bird’s home base station, (i) if the home base station signal strength was stronger than −11.484 (the estimated signal strength at 50 m; see S5 Fig), we assigned the tagged bird the home base station location; (ii) if this was not the case, we assigned the tagged bird the location of the base station with the strongest mean signal strength. (PNG) [file pbio.3002859.s004.png]

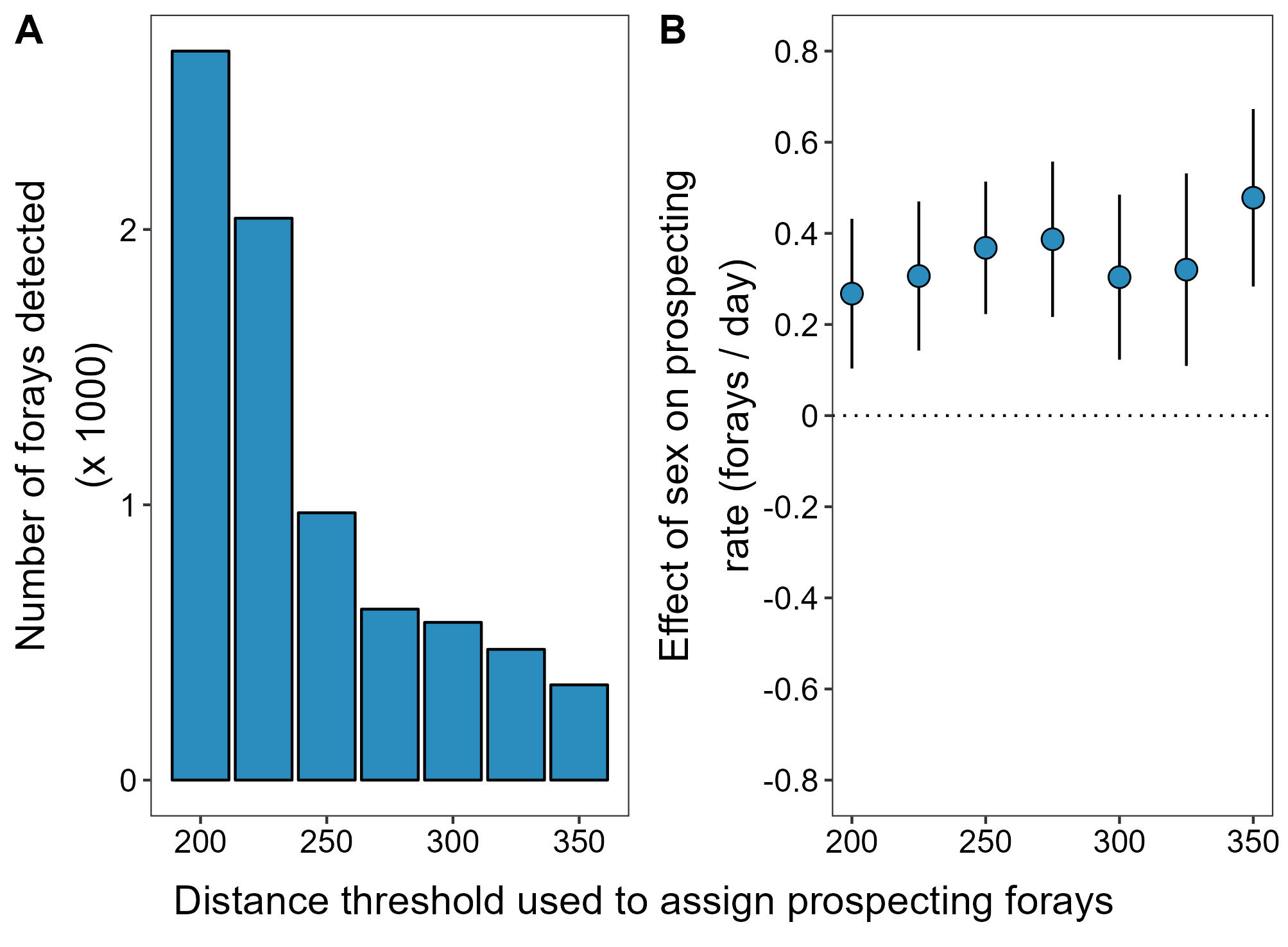

Supplement: S4 Fig — Sensitivity analysis to assess the effect of the “distance threshold” set during the foray detection process (see Methods) on (A) the total number of detected “forays” and (B) the effect size estimate for the sex difference in prospecting rate (forays/day; males relative to females). (A) A “foray” was only considered to have occurred if the base station receiver that the focal bird was estimated to be closest to (i.e., its “best estimate” location) was further than a set distance threshold away from the base station at the centre of the bird’s home territory (see Methods). A priori we set this distance threshold to be 250 m as this approach renders it highly likely that the focal bird itself is >125 m away from the centre of their home territory (see Methods in the main paper for the rationale). As the mean (± SE) distance between the centres of neighbouring territories is 93.7 m (± 4.56 m) in our study population, this minimum plausible distance of 125 m from the centre of the bird’s home territory should ensure that “forays” detected using a 250 m distance threshold will typically have involved movements beyond the territory-centres of the tagged bird’s neighbouring groups. This approach should thereby minimise the chance that the bird’s territorial interactions with its neighbours along their shared boundary while “at home” are misclassified as extra-territorial forays. Reducing the distance threshold below 250 m will progressively increase the risk of such misclassifications; the likely cause of the marked increase in the number of “forays” detected when threshold distances of 225 m and 200 m are used (panel A), while increasing the distance threshold above 250 m may yield an excessively conservative approach that substantially underestimates the incidence of “true forays” by failing to capture those that occur over shorter distances. (B) The effect that changing this set distance threshold has on the estimated effect size (± SE) for the overall sex difference i [file pbio.3002859.s005.png]

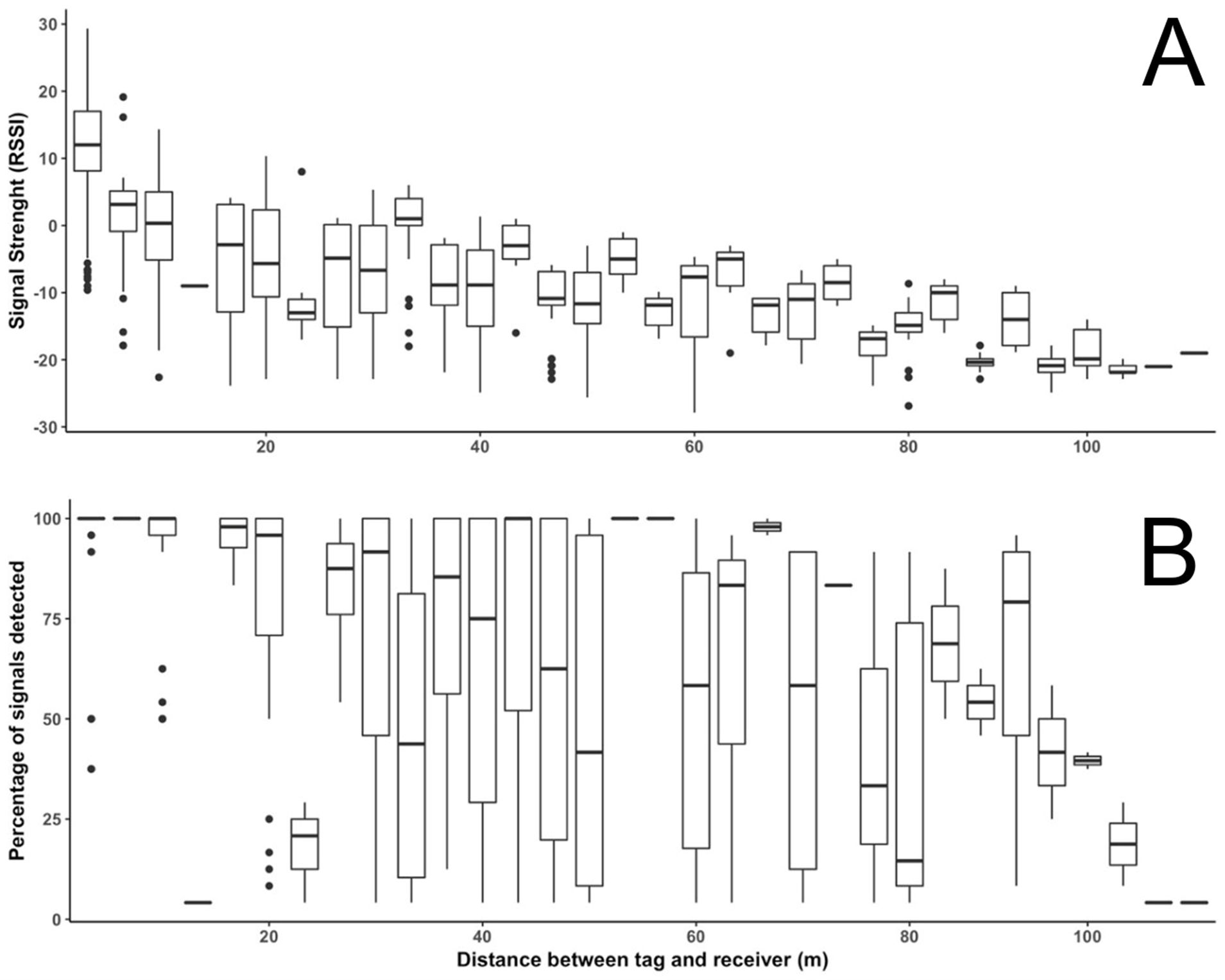

Supplement: S5 Fig — The received signal strength indicator (RSSI) values (A) and percentage of detected signals (B) both decreased with the distance between tags and base-station receivers in a field validation on our study site. Our workflow for using the distribution of signal strengths across our receiver array to allocate “best estimate” locations for the tagged birds in each 15-s window, principally used information on the relative signal strength between receivers whenever tags were detected at multiple receivers simultaneously (see S3 Fig for details). However, to add an additional layer of conservatism to the assignment of “non-home” locations in scenarios in which a tag was detected by the receiver in the centre of the tagged bird’s “home” territory as well as one or more receivers elsewhere, we also sought to estimate a threshold absolute signal strength that, if exceeded by the receiver on the home territory, would act as another indicator that the tagged bird was likely “home.” To do this, we estimated the signal strength (RSSI)-distance relationship within our study site (panel A) using biologically realistic locations for the birds via the method described below, and then calculated the mean signal strength obtained at 50 m distance from a receiver in this context for use as this threshold value (as the mean ± SE distance between neighbouring territory centres is just 93.7 m ± 4.56 m in our study population). This process yielded a threshold RSSI value of −11.484; so if a tagged bird was registered with an RSSI value above −11.484 at the receiver at the centre of its “home” territory, the bird was conservatively assigned a “home” location regardless of the signal strengths logged in other locations (see the final step in the S3 Fig workflow). We appreciate that the inherent variability within the RSSI-distance relationship (panel a; due to the effects for example of variation in tag height and signal obstruction via natural features on the study site) has 2 implications, [file pbio.3002859.s006.png]
