# Supplementary material for: Evolution of sex differences in cooperation can be explained by trade-offs with dispersal
Source: PLoS Biol. 2024 Oct 24;22(10):e3002859. doi: 10.1371/journal.pbio.3002859 (PMC11500963; doi:10.1371/journal.pbio.3002859)
Supplement: S1 Table — The interaction between subordinate age and subordinate sex did not receive statistical support (χ23 = 6.39, p = 0.094) and was dropped from the full model to ease interpretation of single effect predictors. Residual variance = 0.262. (DOCX) [file pbio.3002859.s007.docx]

**S1 Table**. Coefficients and likelihood-ratio tests of Gaussian mixed model explaining variation in the provisioning visit duration of subordinates within their natal groups (response variable, originally in seconds, ln+1 transformed; n = 5,040 provisioning visits by 205 subordinates, 97 males and 109 females). The interaction between subordinate age and subordinate sex did not receive statistical support (χ^2^_3_ = 6.39, p = 0.094) and was dropped from the full model to ease interpretation of single effect predictors. Residual variance = 0.262.

| **Fixed effect** | **Estimate** | **SE*^A^*** | **95% CI*^A^*** | **χ^2^** | **df*^A^*** | **p** | |
| --- | --- | --- | --- | --- | --- | --- | --- |
| **Intercept** | 3.455 | 0.140 | 3.180, 3.729 |  |  |  | |
| **Subordinate sex** |  |  |  | 16.46 | 1 | <0.001 | |
| *Female* | — | — | — |  |  |  | |
| *Male* | -0.171 | 0.041 | -0.252, -0.090 |  |  |  | |
| **Subordinate age (years)** |  |  |  | 8.08 | 3 | 0.044 | |
| *< 1* | — | — | — |  |  |  | |
| *1-2* | -0.055 | 0.043 | -0.139, 0.028 |  |  |  | |
| *2-3* | -0.160 | 0.055 | -0.268, -0.052 |  |  |  | |
| *>4* | -0.049 | 0.101 | -0.247, 0.149 |  |  |  | |
| **Brood age** |  |  |  | 32.75 | 6 | <0.001 | |
| *6* | — | — | — |  |  |  | |
| *7* | -0.041 | 0.092 | -0.220, 0.139 |  |  |  | |
| *8* | -0.271 | 0.100 | -0.467, -0.076 |  |  |  | |
| *9* | -0.193 | 0.100 | -0.390, 0.003 |  |  |  | |
| *10* | -0.236 | 0.099 | -0.429, -0.042 |  |  |  | |
| *11* | -0.275 | 0.099 | -0.470, -0.081 |  |  |  | |
| *12* | -0.304 | 0.099 | -0.498, -0.110 |  |  |  | |
| **Brood size** | -0.095 | 0.050 | -0.193, 0.003 | 3.53 | 1 | 0.060 | |
| **Random effect variance** | **Estimate** | **# Levels** |  |  |  |  | |
| Individual ID | 0.486 | 205 |  |  |  |  | |
| Social group ID | 0.000 | 31 |  |  |  |  | |
| Breeding season | 0.005 | 8 |  |  |  |  | |
| Clutch ID | 0.050 | 123 |  |  |  |  | |
| *^A^* SE = Standard Error, CI = Confidence Interval, df = degrees of freedom likelihood-ratio test. | | | | | | |  |
