# Supplementary material for: Evolution of sex differences in cooperation can be explained by trade-offs with dispersal
Source: PLoS Biol. 2024 Oct 24;22(10):e3002859. doi: 10.1371/journal.pbio.3002859 (PMC11500963; doi:10.1371/journal.pbio.3002859)
Supplement: S2 Table — The interaction between subordinate age and subordinate sex did not receive statistical support (χ23 = 0.31, p = 0.859) and was dropped from the full model to ease interpretation of single effect predictors. Model coefficients are shown in the link-function scale (“logit”). (DOCX) [file pbio.3002859.s008.docx]

**S2 Table**. Coefficients and likelihood-ratio tests of binomial mixed model explaining variation in probability of provisioning a large food item by subordinates within their natal groups (n = 1,325 provisioning visits by 156 subordinates, 74 males and 83 females). The interaction between subordinate age and subordinate sex did not receive statistical support (χ^2^_3_ = 0.31, p = 0.859) and was dropped from the full model to ease interpretation of single effect predictors. Model coefficients are shown in the link-function scale (‘logit’).

| **Fixed effect** | **Estimate** | **SE*^A^*** | **95% CI*^A^*** | **χ^2^** | **df*^A^*** | **p** |
| --- | --- | --- | --- | --- | --- | --- |
| **Intercept** | 0.820 | 1.418 | -1.960, 3.599 |  |  |  |
| **Subordinate sex** |  |  |  | 1.43 | 1 | 0.231 |
| *Female* | — | — | — |  |  |  |
| *Male* | -0.339 | 0.286 | -0.899, 0.222 |  |  |  |
| **Subordinate age (years)** |  |  |  | 3.57 | 3 | 0.312 |
| *< 1* | — | — | — |  |  |  |
| *1-2* | 0.083 | 0.329 | -0.562, 0.728 |  |  |  |
| *2-3* | 0.370 | 0.448 | -0.509, 1.249 |  |  |  |
| *>4* | -15.044 | 114.487 | -239.434, 209.347 |  |  |  |
| **Brood age** |  |  |  | 7.73 | 6 | 0.258 |
| *6* | — | — | — |  |  |  |
| *7* | -3.282 | 1.581 | -6.381, -0.184 |  |  |  |
| *8* | -3.328 | 1.328 | -5.930, -0.725 |  |  |  |
| *9* | -3.409 | 1.296 | -5.950, -0.868 |  |  |  |
| *10* | -3.365 | 1.282 | -5.878, -0.853 |  |  |  |
| *11* | -3.312 | 1.278 | -5.816, -0.807 |  |  |  |
| *12* | -3.386 | 1.282 | -5.899, -0.872 |  |  |  |
| **Brood size** | 0.119 | 0.338 | -0.543, 0.781 | 0.12 | 1 | 0.725 |
| **Random effect variance** | **Estimate** | **# Levels** |  |  |  |  |
| Individual ID | 0.391 | 156 |  |  |  |  |
| Social group ID | 0.396 | 27 |  |  |  |  |
| Breeding season | 0.000 | 7 |  |  |  |  |
| Clutch ID | 0.773 | 87 |  |  |  |  |
| *^A^*  SE = Standard Error, CI = Confidence Interval, df = degrees of freedom likelihood-ratio test. | | | | | | |
