# Supplementary material for: Evolution of sex differences in cooperation can be explained by trade-offs with dispersal
Source: PLoS Biol. 2024 Oct 24;22(10):e3002859. doi: 10.1371/journal.pbio.3002859 (PMC11500963; doi:10.1371/journal.pbio.3002859)
Supplement: S3 Table — This table shows the result of a linear model explaining variation in microsatellite relatedness [5] between subordinates (males and females) and the offspring that they help to rear. In the model subordinate sex was included as a fixed effect predictor. For details of microsatellite genotyping, see Supplementary Text A in [6,7]. Residual variance = 0.041. (DOCX) [file pbio.3002859.s009.docx]

**S3 Table**. Microsatellite relatedness of subordinates and offspring (n = 487 relatedness measures for 205 subordinates, 111 males and 94 females). This table shows the result of a linear model explaining variation in microsatellite relatedness [5] between subordinates (males and females) and the offspring that they help to rear. In the model subordinate sex was included as a fixed effect predictor. For details of microsatellite genotyping, see Supplementary Text A in [6] and [7]. Residual variance = 0.041.

| **Fixed effect** | **Estimate** | **SE*^A^*** | **95% CI*^A^*** | **χ^2^** | **df*^A^*** | **p** |  |
| --- | --- | --- | --- | --- | --- | --- | --- |
| **Intercept** | 0.374 | 0.020 | 0.335, 0.413 |  |  |  |  |
| **Subordinate sex** |  |  |  | 0.16 | 1 | 0.691 |  |
| *Female* | — | — | — |  |  |  |  |
| *Male* | -0.008 | 0.021 | -0.049, 0.032 |  |  |  |  |
| **Random effect variance** | **Estimate** | **# Levels** |  |  |  |  |  |
| Clutch ID | 0.017 | 109 |  |  |  |  |  |
| *^A^* SE = Standard Error, CI = Confidence Interval, df = degrees of freedom likelihood-ratio test. | | | | | | | |
