# Supplementary material for: Evolution of sex differences in cooperation can be explained by trade-offs with dispersal
Source: PLoS Biol. 2024 Oct 24;22(10):e3002859. doi: 10.1371/journal.pbio.3002859 (PMC11500963; doi:10.1371/journal.pbio.3002859)
Supplement: S4 Table — The interaction between subordinate age and subordinate sex did not receive statistical support (χ23 = 0.80, p = 0.850) and was dropped from the full model to ease interpretation of single effect predictors. Model coefficients are shown in the link-function scale (“logit”). (DOCX) [file pbio.3002859.s010.docx]

**S4 Table.** Coefficients and likelihood-ratio tests of binomial mixed model explaining variation in probability of subordinate dominance acquisition in the natal group when subordinates resided in the natal group at 1, 2, 3 and 4 years of age (n = 375 age-specific observations, 114 males and 105 females). The interaction between subordinate age and subordinate sex did not receive statistical support (χ^2^_3_ = 0.80, p = 0.850) and was dropped from the full model to ease interpretation of single effect predictors. Model coefficients are shown in the link-function scale (‘logit’).

| **Fixed effect** | **Estimate** | **SE*^A^*** | **95% CI*^A^*** | **χ^2^** | **df*^A^*** | **p** |
| --- | --- | --- | --- | --- | --- | --- |
| **Intercept** | -4.315 | 1.125 | -6.519, -2.111 |  |  |  |
| **Subordinate age** |  |  |  | 24.76 | 3 | < 0.001 |
| *1* | — | — | — |  |  |  |
| *2* | 0.683 | 0.476 | -0.250, 1.616 |  |  |  |
| *3* | 2.115 | 0.600 | 0.939, 3.292 |  |  |  |
| *4* | 3.598 | 0.945 | 1.745, 5.451 |  |  |  |
| **Sex** |  |  |  | 0.29 | 1 | 0.592 |
| *Female* | — | — | — |  |  |  |
| *Male* | -0.255 | 0.482 | -1.200, 0.690 |  |  |  |
| **Random effect variance** | **Estimate** | **# Levels** |  |  |  |  |
| Social group ID | 3.953 | 35 |  |  |  |  |
| Breeding season of hatching | 0.398 | 6 |  |  |  |  |
| *^A^* SE = Standard Error, CI = Confidence Interval, df = degrees of freedom likelihood-ratio test. | | | | | | |
