# Supplementary material for: Evolution of sex differences in cooperation can be explained by trade-offs with dispersal
Source: PLoS Biol. 2024 Oct 24;22(10):e3002859. doi: 10.1371/journal.pbio.3002859 (PMC11500963; doi:10.1371/journal.pbio.3002859)
Supplement: S5 Table — The interaction between subordinate age and subordinate sex did not receive statistical support (χ23 = 0.03, p = 0.870) and was dropped from the full model to ease interpretation of single effect predictors. Model coefficients are shown in the link-function scale (“logit”). (DOCX) [file pbio.3002859.s011.docx]

**S5 Table.** Coefficients and likelihood-ratio tests of binomial mixed model explaining variation in probability of subordinate dominance acquisition in the natal group when subordinates resided in the natal group at 1, 2, 3 and 4 years of age, including events any in which dominance was acquired outside the natal group by founding a new group within territory previously held by the natal group (i.e., territorial budding [8]) (n = 375 age-specific observations, 114 males and 105 females). The interaction between subordinate age and subordinate sex did not receive statistical support (χ^2^_3_ = 0.03, p = 0.870) and was dropped from the full model to ease interpretation of single effect predictors. Model coefficients are shown in the link-function scale (‘logit’).

| **Fixed effect** | **Estimate** | **SE*^A^*** | **95% CI*^A^*** | **χ^2^** | **df*^A^*** | **p** |  |
| --- | --- | --- | --- | --- | --- | --- | --- |
| **Intercept** | -3.151 | 0.916 | -4.947, -1.355 |  |  |  |  |
| **Subordinate age** |  |  |  | 24.37 | 3 | < 0.001 |  |
| *1* | — | — | — |  |  |  |  |
| *2* | 0.792 | 0.405 | -0.002, 1.587 |  |  |  |  |
| *3* | 1.919 | 0.546 | 0.849, 2.988 |  |  |  |  |
| *4* | 3.146 | 0.822 | 1.534, 4.758 |  |  |  |  |
| **Sex** |  |  |  | 1.89 | 1 | 0.169 |  |
| *Female* | — | — | — |  |  |  |  |
| *Male* | -0.525 | 0.387 | -1.284, 0.234 |  |  |  |  |
| **Random effect variance** | **Estimate** | **# Levels** |  |  |  |  |  |
| Social group ID | 2.033 | 35 |  |  |  |  |  |
| Breeding season of hatching | 2.557 | 5 |  |  |  |  |  |
| SE = Standard Error, CI = Confidence Interval, df = degrees of freedom likelihood-ratio test. | | | | | | | |
