# Supplementary material for: Evolution of sex differences in cooperation can be explained by trade-offs with dispersal
Source: PLoS Biol. 2024 Oct 24;22(10):e3002859. doi: 10.1371/journal.pbio.3002859 (PMC11500963; doi:10.1371/journal.pbio.3002859)
Supplement: S6 Table — No statistical support was found for the interaction between sex and provisioning (χ21 = 0.97, p = 0.324), which was removed from the final model. Model coefficients (Estimate) are shown along with standard errors (SE) and 95% confidence intervals (95% CIs). Model coefficients are shown in the link-function scale (“log”). (DOCX) [file pbio.3002859.s012.docx]

**S6 Table.** Coefficients and likelihood-ratio tests of Poisson mixed model explaining variation in prospecting rate (e.g., number of prospecting forays per day; n = 895 daily measures of prospecting rate from 27 tagged birds). No statistical support was found for the interaction between sex and provisioning (χ^2^_1_ = 0.97, p = 0.324), which was removed from the final model. Model coefficients (Estimate) are shown along with standard errors (SE) and 95% confidence intervals (95% CI). Model coefficients are shown in the link-function scale (‘log’).

| **Fixed effect** | **Estimate** | **SE*^A^*** | **95% CI*^A^*** | **χ^2^** | **df*^A^*** | **p** |
| --- | --- | --- | --- | --- | --- | --- |
| **Intercept** | -0.546 | 0.199 | -0.936, -0.155 |  |  |  |
| **Subordinate sex** |  |  |  | 5.39 | 1 | 0.020 |
| *Female* | — | — | — |  |  |  |
| *Male* | 0.368 | 0.145 | 0.083, 0.653 |  |  |  |
| **Provisioning phase** |  |  |  | 2.15 | 1 | 0.142 |
| *No* | — | — | — |  |  |  |
| *Yes* | -0.144 | 0.094 | -0.328, 0.040 |  |  |  |
| **Subordinate age** | 0.030 | 0.060 | -0.088, 0.148 | 0.23 | 1 | 0.631 |
| **Random effect variance** | **Estimate** | **# Levels** |  |  |  |  |
| Observation ID | 0.432 | 895 |  |  |  |  |
| Individual ID | 0.030 | 27 |  |  |  |  |
| Social group ID | 0.290 | 14 |  |  |  |  |
| *^A^* SE = Standard Error, CI = Confidence Interval, df = degrees of freedom likelihood-ratio test. | | | | | | |
