# Supplementary material for: Evolution of sex differences in cooperation can be explained by trade-offs with dispersal
Source: PLoS Biol. 2024 Oct 24;22(10):e3002859. doi: 10.1371/journal.pbio.3002859 (PMC11500963; doi:10.1371/journal.pbio.3002859)
Supplement: S7 Table — No statistical support was found for the interaction between sex and provisioning (χ21 = 1.14, p = 0.285), which was removed from the final model. Model coefficients (Estimate) are shown along with standard errors (SE) and 95% confidence intervals (95% CIs). Residual variance = 1.819. (DOCX) [file pbio.3002859.s013.docx]

**S7 Table.** Coefficients and likelihood-ratio tests of Gaussian mixed model (with log-transformed response variable) explaining variation in duration of individual forays (log minutes; n = 971 prospecting forays from 27 tagged birds). No statistical support was found for the interaction between sex and provisioning (χ^2^_1_ = 1.14, p = 0.285), which was removed from the final model. Model coefficients (Estimate) are shown along with standard errors (SE) and 95% confidence intervals (95% CI). Residual variance = 1.819.

| **Fixed effect** | **Estimate** | **SE*^A^*** | **95% CI*^A^*** | **χ^2^** | **df*^A^*** | **p** |  |
| --- | --- | --- | --- | --- | --- | --- | --- |
| **Intercept** | 2.405 | 0.288 | 1.840, 2.969 |  |  |  |  |
| **Subordinate sex** |  |  |  | 0.08 | 1 | 0.773 |  |
| *Female* | — | — | — |  |  |  |  |
| *Male* | -0.060 | 0.235 | -0.521, 0.401 |  |  |  |  |
| **Provisioning phase** |  |  |  | 0.07 | 1 | 0.796 |  |
| *No* | — | — | — |  |  |  |  |
| *Yes* | -0.029 | 0.111 | -0.247, 0.188 |  |  |  |  |
| **Subordinate age** | -0.193 | 0.100 | -0.389, 0.002 | 3.95 | 1 | 0.047 |  |
| **Random effect variance** | **Estimate** | **# Levels** |  |  |  |  |  |
| Individual ID | 0.153 | 27 |  |  |  |  |  |
| Social group ID | 0.477 | 14 |  |  |  |  |  |
| *^A^* SE = Standard Error, CI = Confidence Interval, df = degrees of freedom likelihood-ratio test. | | | | | | | |
