# Supplementary material for: Evolution of sex differences in cooperation can be explained by trade-offs with dispersal
Source: PLoS Biol. 2024 Oct 24;22(10):e3002859. doi: 10.1371/journal.pbio.3002859 (PMC11500963; doi:10.1371/journal.pbio.3002859)
Supplement: S8 Table — No statistical support was found for the interaction between sex and provisioning (χ21 = 0.142, p = 0.707), which was removed from the final model. Model coefficients (Estimate) are shown along with standard errors (SE) and 95% confidence intervals (95% CIs). Residual variance = 0.059. (DOCX) [file pbio.3002859.s014.docx]

**S8 Table.** Coefficients and likelihood-ratio tests of Gaussian mixed model (with log-transformed response variable) explaining variation in distance of individual forays (log meters; n = 971 prospecting forays from 27 tagged birds). No statistical support was found for the interaction between sex and provisioning (χ^2^_1_ = 0.142, p = 0.707), which was removed from the final model. Model coefficients (Estimate) are shown along with standard errors (SE) and 95% confidence intervals (95% CI). Residual variance = 0.059.

| **Fixed effect** | **Estimate** | **SE*^A^*** | **95% CI*^A^*** | **χ^2^** | **df*^A^*** | **p** |  |
| --- | --- | --- | --- | --- | --- | --- | --- |
| **Intercept** | 6.563 | 0.046 | 6.472, 6.654 |  |  |  |  |
| **Subordinate sex** |  |  |  | < 0.01 | 1 | 0.970 |  |
| *Female* | — | — | — |  |  |  |  |
| *Male* | -0.002 | 0.040 | -0.080, 0.076 |  |  |  |  |
| **Provisioning phase** |  |  |  | 0.78 | 1 | 0.377 |  |
| *No* | — | — | — |  |  |  |  |
| *Yes* | -0.018 | 0.020 | -0.057, 0.021 |  |  |  |  |
| **Subordinate age** | -0.006 | 0.017 | -0.039, 0.027 | 0.14 | 1 | 0.708 |  |
| **Random effect variance** | **Estimate** | **# Levels** |  |  |  |  |  |
| Individual ID | 0.004 | 27 |  |  |  |  |  |
| Social group ID | 0.010 | 14 |  |  |  |  |  |
| *^A^* SE = Standard Error, CI = Confidence Interval, df = degrees of freedom likelihood-ratio test. | | | | | | | |
