# Supplementary material for: Evolution of sex differences in cooperation can be explained by trade-offs with dispersal
Source: PLoS Biol. 2024 Oct 24;22(10):e3002859. doi: 10.1371/journal.pbio.3002859 (PMC11500963; doi:10.1371/journal.pbio.3002859)
Supplement: S9 Table — The data set comprised 34 daily measures of provisioning rate (each with a paired estimate of prospecting effort) from 13 subordinates (6 males and 7 females) in 7 social groups. Prospecting rate was calculated in the 3 days leading up to the measurement of cooperative provisioning rate. Despite the modest sample size, the model recovered the significant negative main effect of prospecting rate on provisioning rate that is expected under the Dispersal trade-off hypothesis. The effects of subordinate sex, brood age, and brood size were included as fixed effects only to ensure that uncontrolled independent effects of these variables were unlikely to be confounding the prospecting effect of interest. Given our use of a conservative full model approach throughout, these terms were retained in the model regardless of their significance. With regard to the interpretation of the effect of subordinate sex, see footnote B. Model coefficients (Estimate) are shown along with standard errors (SEs) and 95% confidence intervals (95% CIs). (DOCX) [file pbio.3002859.s015.docx]

**S9 Table.** Poisson mixed model testing for a negative effect of prospecting rate (forays / day) on cooperative provisioning rate (feeds / hour). The data set comprised 34 daily measures of provisioning rate (each with a paired estimate of prospecting effort) from 13 subordinates (six males and seven females) in seven social groups. Prospecting rate was calculated in the three days leading up to the measurement of cooperative provisioning rate. Despite the modest sample size, the model recovered the significant negative main effect of prospecting rate on provisioning rate that is expected under the Dispersal trade-off hypothesis. The effects of subordinate sex, brood age and brood size were included as fixed effects only to ensure that uncontrolled independent effects of these variables were unlikely to be confounding the prospecting effect of interest. Given our use of a conservative full model approach throughout, these terms were retained in the model regardless of their significance. With regard to the interpretation of the effect of subordinate sex, see footnote B. Model coefficients (Estimate) are shown along with standard errors (SE) and 95% confidence intervals (95% CI).

| **Fixed effect** | **Estimate** | **SE*^A^*** | **95% CI*^A^*** | **χ^2^** | **df*^A^*** | **p** |  |
| --- | --- | --- | --- | --- | --- | --- | --- |
| **Intercept** | -2.321 | 1.356 | -4.979, 0.337 |  |  |  |  |
| **Prospecting rate (forays/day)** | -0.367 | 0.165 | -0.691, -0.043 | 5.08 | 1 | 0.024 |  |
| **Brood size** | 0.415 | 0.357 | -0.286, 1.115 | 1.31 | 1 | 0.253 |  |
| **Brood age** | 0.201 | 0.099 | 0.008, 0.395 | 4.13 | 1 | 0.042 |  |
| **Subordinate sex** |  |  |  | 1.93 | 1 | 0.165 |  |
| *Female* | — | — | — |  |  |  |  |
| *Male* | -0.711 | 0.509 | -1.709, 0.287 |  |  |  |  |
| **Random effect variance** | **Estimate** | **# Levels** |  |  |  |  |  |
| Individual ID | 0.471 | 13 |  |  |  |  |  |
| Social group ID | 0.000 | 7 |  |  |  |  |  |
| *^A^*  SE = Standard Error, CI = Confidence Interval, df = degrees of freedom likelihood-ratio test.  *^B^* While this lack of a significant effect of subordinate sex on cooperative provisioning rate (when simultaneously controlling for the effect of prospecting) also aligns with expectations under the Dispersal trade-off hypothesis, this result should be interpreted with caution as the power of this analysis to detect sex differences is likely low (data available for just six males and seven females). | | | | | | | |
