# Supplementary material for: Evolution of sex differences in cooperation can be explained by trade-offs with dispersal
Source: PLoS Biol. 2024 Oct 24;22(10):e3002859. doi: 10.1371/journal.pbio.3002859 (PMC11500963; doi:10.1371/journal.pbio.3002859)
Supplement: S10 Table — Model coefficients are shown in the link-function scale (“logit”). (DOCX) [file pbio.3002859.s016.docx]

**S10 Table.** Coefficients and likelihood-ratio tests of binomial mixed model explaining variation in the probability that natal subordinate individuals emigrate to a subordinate position (n = 88 dispersal events; 46 of 56 observed natal male dispersals; 19 of 32 observed natal female dispersals). Model coefficients are shown in the link-function scale (‘logit’).

| **Fixed effect** | **Estimate** | **SE*^A^*** | **95% CI*^A^*** | **χ^2^** | **df^A^** | **p** |  |
| --- | --- | --- | --- | --- | --- | --- | --- |
| **Intercept** | 0.589 | 0.495 | -0.380, 1.559 |  |  |  |  |
| **Sex** |  |  |  | 4.16 | 1 | 0.041 |  |
| *Female* | — | — | — |  |  |  |  |
| *Male* | 1.063 | 0.520 | 0.045, 2.081 |  |  |  |  |
| **Random effect variance** | **Estimate** | **# Levels** |  |  |  |  |  |
| Social group ID | 0.000 | 30 |  |  |  |  |  |
| Breeding season of hatching | 0.266 | 7 |  |  |  |  |  |
| *^A^* SE = Standard Error, CI = Confidence Interval, df = degrees of freedom likelihood-ratio test. | | | | | | | |
