# Supplementary material for: Evolution of sex differences in cooperation can be explained by trade-offs with dispersal
Source: PLoS Biol. 2024 Oct 24;22(10):e3002859. doi: 10.1371/journal.pbio.3002859 (PMC11500963; doi:10.1371/journal.pbio.3002859)
Supplement: Attachment — Submitted filename: Response to Reviewers - PBIOLOGY-D-24-01513R1.pdf [file pbio.3002859.s017.pdf]

Point-by-point response to reviewers' comments - PBIOLOGY-D-24-01513R1

Reviewer #1:

This is a nice paper arguing persuasively that the unusual pattern of female sex bias in provisioning behavior observed in the cooperatively breeding white-browed sparrow weaver is a consequence of a trade-off between the time subordinates spend looking for opportunities to disperse from their natal group and the time they have to feed young rather than that females are more likely to gain direct fitness advantages by inheriting dominance status in their natal group. The author's arguments are not iron-clad, but they're good and the extensive long-term data that they have obtained on this interesting species offers a strong argument supporting the "dispersal trade-off" hypothesis. In particular, their radio-tracking data, made possible by recent advances in tracking technology, provides novel a insight into this hypothesis. The authors do a good job of pointing out the potential wider implications of their results in that they counter the recent trend for claiming that direct fitness benefits play a widespread role in the evolution of helping behavior.

> Authors' Response: Thank you for the time taken to read our manuscript and for your thorough and constructive feedback. We have addressed all comments raised (see below for point-by-point responses). In doing so, we believe that the revised version of the manuscript is clearer than the initial version.

Aspects of the authors' arguments that are worth additional discussion include:

1) The female bias in provisioning that they observe is indeed unusual, but largely because helpers in many species of cooperative breeders are primarily (if not exclusively) male. I don't know offhand whether this is also true for species in which both males and females serve as helpers relatively commonly; the authors should check.

> Authors' Response: Thank you for this comment, which is correct. We have now inserted two new sentences in the discussion to clarify this point (L319-324):
>
> "*While our finding of female-biased natal cooperation is unusual for cooperative birds, this is principally the case because in many cooperative bird species only males delay dispersal and so only males are available to help within the natal group [2]. Among those cooperative bird species in which both sexes delay dispersal from their natal group and help, significantly female-biased rates of helping with one or more forms of care while within the natal group now appear to have been documented in a comparable number of species to significantly male-biased rates of helping [4].*"
>
> We have also nuanced our treatment further by removing the term 'rare' from our previous description of this reversal of the avian norm for male-biased cooperation (final paragraph of the introduction and first paragraph of the discussion), as the extent to which this is rare depends on how one examines patterns of cooperation (as our new text above makes clear). We retain the use of 'rare' only to describe the discovery here of male-biased dispersal in a bird, which is certainly rare.

(They may have said this, but is the propensity for males and females to be subordinates the same; i.e., is the sex ratio of subordinates basically 50:50?)

> Authors' Response: Indeed, our previous work does suggest that the subordinate sex ratio is approximately 1:1 (see Fig. 1b in Capilla-Lasheras et al., 2021). We have included this point in the Introduction (L115-116).

2) The authors' argument regarding the greater effect of female subordinates is based on behavior, not fitness consequences. They do mention some aspects of the latter, but for the most part they say little about the role that subordinates play in enhancing reproductive success of groups or survivorship of dominants. The latter is potentially important in that if there is a difference it could result in significantly different future indirect fitness benefits of subordinate males compared to females.

Authors' Response: We appreciate the point and have now added further detail to our original coverage of the fitness consequences of helping in this system (L133-146; see our reply to the comment specifically about this below).

With specific regard to the possibility of a sex difference in the indirect fitness benefits of helping (r x b in Hamilton's rule), it is not clear how such a sex difference could arise without a sex difference in the relatedness of the actor to the recipient (r; which we have demonstrated is not the case here; Figure 1E), as this would require a sex difference among actors in the benefit that a given unit of helping provided to the recipient (i.e., in b in Hamilton's rule). For example, in this case, it would require that a given unit of provisioned food has a greater beneficial effect on the chicks (and/or their parents) if it is provided by a female helper than by a male helper, and it is not clear why this would be the case. This is why arguments about the potential for sex differences in indirect fitness benefits focus on the lack of a sex difference in r(elatedness) rather than the possibility of a sex difference in b (e.g. see Downing et al., 2018 for similar arguments). We have now inserted an additional sentence in the discussion to clarify this point (L337-341).

The point about possible sex specific implications of improving dominant survivorship is still an interesting one though, as while the indirect benefits of doing so seem unlikely to differ between the sexes (as explained above), it is conceivable that the sexes differ in the direct fitness costs of doing so. We have therefore included additional text in the discussion to acknowledge this possibility and to explain why it seems unlikely that this could account for our findings (L362-370).

3) The differences in provisioning that they focus on also has potential relevance to the sex ratio via the repayment hypothesis (e.g. Emlen et al. 1986. Am. Nat. 127, 1-8). I don't encourage the authors to go down this rabbit hole, but it might be worth mentioning and is at least something the authors might want to look into at a later time.

Authors' Response: Thank you for this suggestion and we do appreciate the point. If it is okay, it would be our preference to avoid complicating the discussion with this point, as (aside from this being beyond the scope of this study) the limited support to date for the repayment hypothesis leaves it arguable as to whether one currently would predict that mothers should adjust sex allocation according to sex differences in helping.

4) I am concerned about the authors' claim that the dispersal trade-off hypothesis is supported by the sex difference in prospecting they observed even when birds are not provisioning nestlings. On the contrary, doesn't this suggest that some entirely different mechanism may be driving the difference in prospecting other than a trade-off with natal cooperation?

Authors' Response: The dispersal trade-off hypothesis proposes that the sex difference in prospecting drives the sex difference in cooperation. Our argument, therefore, is that if the observed association between the two traits was driven instead by the sex difference in cooperation driving the sex difference in prospecting (the reverse causation to that proposed in the hypothesis), one might expect to see a sex

difference in prospecting *only* during periods of cooperation, yet we see it outside periods of cooperation too. We made this point in L412-415 and would be happy to clarify further if required. The reviewer is absolutely right that we do not know what is driving the sex difference in prospecting, but evolutionary explanations for this lie outside the scope of this study.

5 I am aware of the difficulties of acquiring and dealing with the radio-tracking data that the authors are using to test the key prediction of the trade-off hypothesis. Despite the relatively modest sample size they were able to gather, their data is nonetheless valuable and important.

Authors' Response: Thank you very much for appreciating the dataset and the manuscript.

Specific comments:

Line 121. The female bias is, of course, not the case is a few avian taxa (such as waterfowl); perhaps this would be better stated as "a rare reversal of the typical passerine sex-bias in dispersal"

Authors' Response: We have made this adjustment as requested (L122).

Line 129. I presume the authors are claiming that "female sparrow-weavers cooperatively feed offspring at substantially higher rates than subordinate males" primarily because female helpers are often rare or absent in cooperative breeders? If this assumption is correct, what about species where both males and females act as helpers? Is this statement still true? Are there more males subordinates that female subordinates?

Authors' Response: We have now clarified in the introductory text (L115-116) that male and female helpers are both common and occur with approximately equal frequency in white-browed sparrow weaver groups. Thus, females contribute more to helping than males not because they are more likely to remain as helpers but because when they do provision at higher rates (as we show here in Figure 1B).

Lines 131-134. Does feeding by subordinates increase reproductive success? Perhaps enhance survival rates by dominants? (In which case it would increase future indirect fitness.)

Authors' Response: We have now added further detail to our description at this location of the impact of helpers on recipients (now L133-146). With regard to the point about the potential indirect fitness implications of this, we have covered this point in our reply to comment 2 above.

Lines 199-201. I realize that individual variation is not the focus of this paper, but were birds that fed more at the nest more likely to remain in their natal group and inherit dominance status?

Authors' Response: We appreciate the interest in this possibility, but answering this question would require a series of additional bespoke analyses as it sits outside the predictions of the focal hypotheses under test in this paper. Given the weight of analyses already in the paper, we would prefer to not detract from an already complex narrative by adding additional analyses to address this point.

Lines 202-205. What advantage to birds gain by dispersing to another group as a subordinate? Why not just stay in their natal group?

Authors' Response: Dispersal offers outbreeding opportunities and may offer immediate access to a dominant position. We have now included additional text in the introduction to make this clear (L118-120).

Line 221. Are the differences still significant if individual birds (rather than forays) are considered independent points? (i.e., the bird was observed to foray or not). This seems like a fairer way to analyze these data.

Authors' Response: The presented test shows that the incidence of observed forays differs from a 50:50 sex ratio. Whether this difference then arises from more males prospecting than females (the suggested additional test) or instead from males and females being similarly likely to engage at all in prospecting but those males that do engage showing higher rates of prospecting than those females that do, is a second question, the answer to which is not yet clear from this analysis. Conducting the suggested additional analysis suggests that there is no sex difference in the *incidence* of prospecting (Binomial test, p = 0.142). Thus, it indicates that the sex difference in prospecting lies more in the *rate* at which males and females prospect than in the likelihood that they ever prospect (which fits with our findings using automated tracking).

Line 252. How is the test mentioned here ("whether the bird's social group was provisioning nestlings at the time" differ from the "nestling provisioning period" on line 250? The statistic cited here ("chi-square=0.97, p=0.32") does not match anything in Table S8.

Authors' Response: Our apologies, the latter statistic is indeed quoted in Table S6 (previously Table S8) but it is in the legend (as it is for a non-significant interaction) rather than the body of the table. We have now clarified this by referring in the results to the table legend rather than just the table (L279).

The statistics reported on L277 and L279 (previously L250 L252) differ because the former refers to the main effect of whether the bird's social group was provisioning (reported in main body of Table S6), while the latter refers to the interaction between this term and the sex of the feeder (reported in the legend of Table S6). Our reference to the legend should now help to clarify this.

Lines 258-264. These are indeed modest sample sizes, but I sympathize with the difficulty of acquiring such data and the authors have done a good job of qualifying what they have.

Authors' Response: Thank you for appreciating the difficulty of collecting and the value of this dataset.

Line 304. It is not strictly true that a lack of any difference in average relatedness to recipients means that differences cannot be potentially attributed to indirect fitness effects; the authors have avoided discussing effects of helping on reproductive success of groups or (more cogently) survivorship of dominants. If helping results in increased survivorship of (related) dominants, females may be gaining future indirect fitness benefits à la Mumme et al. (1989, Helping behaviour, reproductive value, and the future component of indirect fitness. Animal Behaviour 38: 331-343.)

Authors' Response: We have now clarified this point by (i) improving our coverage of the impact of helping on recipients in this system within the introduction (L133-146), (ii) explaining why there is not likely to be a sex difference in the indirect fitness benefits of helping (L337-341), and (iii) acknowledging the possibility that impacts on dominant survivorship do have the potential to yield sex differences in direct fitness costs of helping, while explaining why we think these are unlikely to account for our findings (L362-370). Our response to the reviewers point 2 above covers these changes in more

detail. We feel that these revisions cover this point well but would be happy to make further adjustments if desired.

Lines 307-309. This implies that there is more than one dominant in a group? Or is there extra-group paternity, but only by dominants (as per lines 125-126)?

Authors' Response: Thanks for highlighting the potential confusion here. It is the latter: there is only one dominant male per social group and dominant males lose paternity to dominant males of other groups. We have amended the text to clarify this point (L345).

Lines 343-344. This appears to counter the argument (mentioned above) that helping might enhance breeder survivorship (or reproductive success?); if so, why do subordinates bother to provision at all (ever)?

Authors' Response: In this passage, we refer only to it being unlikely that helping yields direct benefits by augmenting group size (because helping does not appear to increase mean offspring survival or the breeding rate of the dominant female). Helping does, however, have the potential to yield indirect fitness benefits because (as we highlight in the Introduction) it may reduce the environmentally induced variance in the reproductive success of breeders, may increase breeder survival and may improve offspring quality. By expanding our coverage of these points in the introduction (L133-146) this should now be clearer.

Lines 369-370. It's unclear to me why the authors seem to feel that the dispersal trade-off hypothesis is supported by the sex difference in prospecting being observed even when birds are not provisioning nestlings. To me, this suggests the possibility that some entirely different mechanism may be driving the difference in prospecting other than a trade-off with natal cooperation.

Authors' Response: Thank you for the opportunity to clarify this point. We have responded to this comment above (point 4). To reiterate, in this manuscript, we aim to investigate the hypotheses for the evolution of the sex difference in cooperation. We do not aim to test hypotheses about the evolutionary origin of the sex difference in prospecting behaviour.

Line 675. Delete "editors".

Authors' Response: Thank you for point this out. Deleted as suggested (L736).

Fig. 2. I don't understand why the solid points are all way above where all the (obvious) translucent points are, especially for older subordinates.

Authors' Response: The solid points in Fig 2 represent model predictions (i.e., the predicted means) for the probability of dominance acquisition. The translucent points represent the raw data points, which in this binomial model are either '1' (i.e., dominance acquired) or '0' (i.e., dominance not acquired). To our eye the solid points (the predicted means) sit between the translucent points at 1 and 0 rather than above them, just as one would expect.

Tables S12 and S13 are mirror images of each other; authors should pick one and delete the other. (Then again, it's a supplement, so maybe it doesn't really matter.)

Authors' Response: We have removed Table S13 following this suggestion.

Thank you for your thorough revision of our manuscript.

Reviewer #2:

I am very pleased with this manuscript in every respect, and I would definitely rate it within the top 10% of manuscripts that I get to review. The research question is interesting and clearly explained, the empirical study is impressive and of the latest state of the art, the data analysis is remarkably careful and detailed, and the conclusions clearly follow from the data. The study provides strong support for the "Dispersal trade-off hypothesis" and thereby makes an important contribution to the research field. Moreover, the text is very well written and easy to understand. This leaves me with only a few relatively minor suggestions that I can make:

> Authors' Response: Thank you very much for your positive feedback and for your suggestions. We have taken your suggestions on board and think they have increased the clarity and reporting quality of the manuscript.

(1) For all mixed-effect models presented in the Supplementary Tables, please add a section of the table that covers all information about the random effects. I suggest to list each random effect (and the residual, where applicable), the size of the variance component, and the number of levels of the random factor. Finally, the legend should always give the number of rows in the analyzed data set. This would ensure that all relevant information can be found in one place (you could then consider shortening the details about number of levels of random effects in the Methods or Legends). This would also make it clear where an observation-level random effect (OLRE) was fitted to account for overdispersion (e.g. S8).

> Authors' Response: Thank you for these suggestions. We have now implemented all of them. In our supplementary tables, we now detail the random effects of each model, their variance component and their number of levels, along with information on the sample size and residual variance.

(2) For Table S8 it is stated that an OLRE was fitted to account for overdispersion in Poisson counts. In Table S11, you either may have forgotten to mention the use of an OLRE, or the count data was not overdispersed. Otherwise, I am a bit worried that the p-values in this Table might be incorrect (see Knief & Forstmeier 2021 in Behavior Research Methods).

> Authors' Response: Thank you for highlighting the lack of clarity here. The model that we present in Table S9 (previously Table S11) did not show evidence of overdispersion (Dispersion test in DHARMa R package test: dispersion = 0.81, p = 0.780) and, therefore, we did not include a OLRE in it. We have clarified this point in the Methods sections (684-685).

(3) In Figure 1D, the estimates close to 0.55 seem to be far off from the data, since the density of raw data points at zero seems to be far greater than the density at 1. Therefore, the estimates should be far below 0.5. Also, the size of the SEs seems unrealistically large. Is this just an error during plotting, or is it that the plotting of model estimates does not work properly in a binomial mixed-model, especially when the random effects are highly influential? In the latter case, how about trying a Gaussian mixed model just for the purpose of plotting estimates that match the mean value of the data (see Knief & Forstmeier 2021)? If that advice turns out useful here, you may also want to double check other binomial models (e.g. Fig. 1A) for that issue.

> Authors' Response: Thank you for pointing this out. There was indeed an error in the script that produced the plot, model predictions were not correctly calculated. We have corrected the error and now the plot looks much more like you predicted, with lower model predictions (see updated Fig. 1). The correction of this plotting issue doesn't affect the inference from the associated (correct) analysis, which was that there was no significant difference between the sexes in this figure.

Minor typos:
(1) Line 144: I think this should be "accompanied by female-biased natal cooperation"

> Authors' Response: Thanks for pointing that out. We have corrected the sentence (L152).

(2) Y-axis of Fig. 1C: replace "log" with "ln". Likewise in Table S3 and in Line 569. Note that R uses the natural logarithm (ln) by default when using the "log" function.

> Authors' Response: Thanks for pointing that out. We have made these suggested corrections.

(3) Line 476: "2.63.1%" contains a typo

> Authors' Response: Thanks for pointing that out. We have corrected the typo (L523).

Reviewer #3:

Overall I enjoyed reading this manuscript.

> Authors' Response: Thank you for your positive feedback and all your suggestions of improvement. We have addressed all comments (see point-by-point response below) and in doing so, we believe the manuscript is now clearer and stronger.

There was some information left out of the introduction that was well covered in the discussion. Nevertheless, I do feel it should be added to the Introduction to increase clarity (see comments below). The study is interesting and overall robust. There are just a couple of issues of concern for me that may affect results and conclusions. Mainly: prospectors were tracked, these data seem good. However, conclusions were made about these movements without knowing how much non-prospectors move. Can we be sure that prospectors move more and have less time available to feed? This is assumed but no data given.

> Authors' Response: Thank you for this comment. We captured and tagged subordinate birds while roosting within their natal groups (i.e., not while prospecting) and we tagged them not knowing whether they were birds that conducted prospecting forays or not (i.e., we did not tag a subset of subordinates that are known to prospect). Thus, the movement patterns of the birds studied in the tracking project is likely to be representative of natal subordinates of their sex and age (i.e., unbiased with respect to whether they are prospect or not). We have now included additional clarification of this point in a STRANGE statement (Webster & Rutz, 2020) (L568-575). Further to this clarification, we have checked our use of the term 'prospectors' in the main text to avoid this causing confusion.

Second, much is made of prospectors feeding young less, but the data appears biased here: provisioning rates were based on visits to the nest using nest observations overall, not when the individual was available to visit the nest. I have explained this in more detail below: but the males may in fact be feeding as much as the females at the times they are present. Not taking this into account will lead to a negative relationship between prospecting and feeding that may not be present.

> Authors' Response: The time entailed in conducting prospecting forays is indeed time that can't be spent feeding young; the two activities are mutually exclusive. These potential 'time costs' of prospecting are one potential mechanism by which the (hypothesised and detected) trade-off between prospecting and cooperative care could arise. We highlight this point in the introduction (now L90-92):
>
> "Investments in prospecting could trade-off against natal cooperation because *the two activities cannot be carried out at the same time, and because costs and physiological changes associated with prospecting could reduce the expression of helping [9,12,14-16]*".
>
> As the time spent prospecting is one potential mechanism by which the trade-off could arise, it is important to not adjust provisioning rates by time spent prospecting, as doing so would lead to an underestimation of the trade-off. That said, we suspect that the time costs *alone* are unlikely to account for the observed trade-off, because the rates and durations of the detected forays are not sufficiently high to account for the rate differences in feeding. We previously made a similar argument in the discussion with respect to energetic costs, so have now expanded this material to clarify that this same rationale applies to time costs too (see L424-425). Even if the time costs alone were sufficient to account for the trade-off, this would be entirely compatible with the dispersal trade-off hypothesis and hence the conclusions of our manuscript (i.e., that a trade-off

between prospecting and cooperation may be a key driver of the observed sex difference in the rates of cooperative nestling feeding).

L 57-58: while it is true in some cooperative spp that one sex contribute more to care than the other, in other cooperative spp this is not the case, and should be acknowledged as such here to avoid this sounding like a general rule.

Authors' Response: Thank you for this suggestion. We have amended this sentence to cover this point by noting the existence of exceptions (L57).

L 75: an additional potential explanation is that they may need to help in line with the pay-to-stay hypothesis

Authors' Response: Thank you for this suggestion and we agree. The intention at this point in the text is not to provide a complete list of the potential mechanisms by which such direct fitness benefits could arise (hence starting the sentence with 'For example'; L71), as doing so would unnecessarily complicate the narrative. We do then cover this possibility among others in the discussion (L333-370 and L391-395), and so would prefer to leave its treatment there, instead of expanding this point in the Introduction.

L 89" suggest change the word 'offspring' here - it assumes groups are simple family groups. In many species, they are complex groups of adults of varying relatedness that may decide to disperse or not, thus offspring is not always correct.

Authors' Response: Following this suggestion, we have changed 'offspring' by 'individuals' (L88).

L 107: unclear what is meant here by 'acting as required'

Authors' Response: We have adjusted the phrasing here to clarify the point (from 'acting as required' to 'acting in the manner that would be required'; L106).

L 121: recent studies have found that in a number of avian species, the previously considered 'typical' sex biases in dispersal/philopatry do not occur. Instead, which sex disperses depends on sex ratios and opportunities in the larger population as a whole. See for example Speelman et al Anim Behav 208, 19-29, 2024, among others.

Authors' Response: We do appreciate the possibility of temporal and spatial variation in dispersal patterns. Here we are talking about the nature of the sex difference in dispersal detected in our study population so far (i.e., that which is relevant to explaining the sex difference in cooperation in our population so far), and our statement is already cautiously phrased "genetic analyses and field observations suggest that males disperse further than females from their natal to breeding sites and thus that females may be the more philopatric sex". As such, it would be our preference to not interrupt the narrative at this point by noting that this might be different if assessed in a different population or at a different time (which could be true of any traits assessed in natural populations).

L 129-131: where do you demonstrate this? There is no citation? Are you referring to the results of this paper, in the introduction?

Authors' Response: Yes, indeed, in this paper. We have inserted "(see results for both findings)" to clarify this (L132). Referring briefly to these initial results at this point allows us to provide a clearer overview for the reader of the structure of the study.

L 134-136: helps can also gain benefits of young surviving by greater predator detection in larger groups, defense of good quality territory etc that leads to greater survival - the focus here seems too narrow in terms of benefits

> Authors' Response: Yes, indeed, we appreciate that helping to augment group size could yield benefits via several mechanisms, and we deal explicitly with this possibility (including the suggestion here that subordinates might gain interim survival or foraging benefits that don't require the inheritance of breeding positions) in the discussion (L384-394). To avoid working through the full array of potential direct fitness benefits that are possible in this system in the Introduction too, we have instead included the following text to highlight (i) that they are collectively unlikely to be substantial, and (ii) that we will deal with this in the discussion (L141-143):
>
> "*Helping could also conceivably yield direct fitness benefits, but as subordinate immigrants rarely help at all [27], it seems unlikely that these benefits are substantial (see discussion for more detail).*"
>
> To avoid repeating this material within the Introduction, we would prefer to retain this existing approach.

L 144: missing word.

> Authors' Response: We have corrected this sentence. Thanks for pointing this out.

L 160: Main results should be in the main body text, not supplemental material: reader has to refer to SM to see the table output for this result.

> Authors' Response: As it stands, the main manuscript results text contains the χ^2 and p values for all results coupled with extensive figures and references to the relevant supplementary tables. As requested, we have now also moved the tables for the two headline analyses of sex differences into the main text; dispersal (now Table 1) and cooperation (now Table 2). As we have a very large number of analyses and associated model tables, we are conscious of the impacts on space of moving more tables in from the supplementary materials.

L 166: but is this relative to opportunity: did they feed more simply because they were there more often? Whereas on a per hour present basis, there was no difference? The figures suggest a prov rate per hour, but was this based on hours observing the nest, or hours that the helpers were present and able to provision young?

> Authors' Response: See above for our reply to the second point. These are simply the unadjusted rates of cooperative feeding at the nest, as it is the sex difference in this trait that the proposed hypotheses seek to explain. It is indeed conceivable that the time taken to prospect is one factor contributing to the observed trade-off between prospecting and cooperative feeding rate (which is fine, as that is one of several mechanisms that could act in isolation or concert to generate a trade-off between prospecting and cooperation; see above and the Introduction). However, we do think it is unlikely that such time costs alone can explain the trade-off observed (see above and the Discussion).

L 166-176: why is it called cooperative provisioning (which suggests it occured with another adult), and not just provisioning? Part way through it does change to just provisioning, and hence this is confusing.

Authors' Response: To clarify that we were merely referring to the provisioning rates of subordinates (which are cooperative as they are helping to rear the offspring of others), we have just removed the terms 'cooperatively' and 'cooperative' from the paragraph and just made clear instead that we are talking about the provisioning rates of subordinates (L174-184).

L 191: this raises the question of why there are sex differences in dispersal then, if females are no more likely to inherit? Can males gain EGP?

Authors' Response: Indeed, we were struck by this point too and address it in the final sentence of the paragraph via reference to a dedicated paragraph of discussion in the supplementary text (L228-231):

"*The observed male-bias in age-specific dispersal probability (Figure 1a) appears, therefore, to reflect a sex difference in the strategy used to acquire dominance via dispersal, rather than in the incidence of acquiring dominance via dispersal (see Supplementary text 4).*"

We do not think that it is driven by patterns of extra-group paternity, as the vast majority of extra-group paternity in this species is won by dominant males.

L 221: but is your adult sex ratio 1:1? If not, then this is a moot point.

Authors' Response: Indeed, our previous work suggests that the subordinate adult sex ratio (that which is relevant to this comparison) is approximately 1:1 (see Fig. 1b in Capilla-Lasheras et al., 2021). We now make this clear in the introduction (L115-116) and have also included a citation to this paper at this point in the results text (L246). We explicitly acknowledge at this point in the text too that this comparison cannot be relied upon in isolation and that this is why our radio-tracking study is important (which then separately confirms this pattern of male-biased prospecting using directly estimated rates of prospecting per individual).

L 235-237: but how different is this movement from typical (non-prospecting) movement in this species. Did you radio track non-prospectors to confirm they were not travelling similar distances (as in, territory boundaries are not stable in many species, and individuals can range widely throughout the day)?

Authors' Response: Please see our reply to the first point raised above. As explained there, we tracked subordinates without regard to the frequency of their prospecting, and so were not only tracking subordinates that do prospect (or neglecting a subset of subordinates that don't prospect, if such a subset exists). We have clarified this point in the Methods (L568-575).

Additionally, from extensive observations of foraging groups we know that the groups are strictly territorial during the breeding season (when all observations in this paper were carried out), maintaining clear boundaries between mutually exclusive foraging territories. The movements documented here as forays are therefore quite distinct from the daily foraging movements of birds within their groups.

L 239: 26 forays a day suggests this species may typically range widely, per above statement

Authors' Response: 26 is the maximum daily rate estimate for prospecting within the entire data set (and so reflects just 1 bird on 1 day). The mean rate of forays was close to 1 foray per day. Please see our reply to the previous comment too, in which we explain

that the 'typical' daily foraging activities of these birds do not involve forays of this kind. The forays were conservatively characterised in a manner that should reflect movements well beyond the boundary of their foraging territory (L550-554).

L 247-257: it is difficult to follow how these results were obtained, since a stats statement is given that refers to various models in SM, and some of the are the same table, some are different tables. For example, in this section, the results reported come from Tables S8, S8, S8, S9,S10,S9,S8,S10. This is very confusing jumps between model tables.

Authors' Response: We appreciate that this section reports a lot of results, but presenting them in an efficient and linear narrative requires that we occasionally refer back to individual results from model tables already cited. The alternative approach of reporting all results from one table before moving on to report all results from the next would be more repetitive and would not highlight linked results (e.g., with the current approach we are able to report the age effects from all three tables together). We are very happy to adjust the text though if there is a clear preference to do so.

L 263-264: it's hard to understand what is meant here. A small sample size that was then varied between potential time windows based on what biological assumptions about appropriate time periods?

Authors' Response: The sample size was not varied, we simply verified that our result was robust to the choice of time window over which each bird's prospecting rate was calculated (given that the most biologically appropriate time window cannot be known and so should not be assumed). So, to investigate the trade-off between prospecting and cooperative provisioning at the individual level, we calculated prospecting rate in a time window prior to the days on which cooperative provisioning was observed, predicting a negative association. Since it is not clear *a priori* over what time window prospecting effort would impact cooperative provisioning (if indeed it does), we tested that the negative association detected was robust to calculating prospecting effort over different time windows, to ensure that any finding was not driven by making an arbitrary assumption about the time window. This rationale is covered in the final paragraph of the methods (L696-701).

L 294: spelling - underling

Authors' Response: Thanks for pointing this out. We have corrected the typo (L326).

L 309: are these extra group males? Or intragroup? Further down in methods this is explained, but at this point in the manuscript, the reader does not know, so would be good to clarify to avoid ambiguity.

Authors' Response: Thanks for pointing out the potential for confusion here. We have clarified the text (L345).

L 375: see note above: this depends on if provisioning rate was calculated as number of hours nest observed, or number of hours individuals was present to provision the nest. If the latter not used, this biases the data

Authors' Response: In this study, we are testing hypotheses that seek to explain sex differences in the absolute rates of cooperative provisioning at the nest. As such, it is appropriate to calculate provisioning rate over the number of hours a nest was observed (doing so does not bias the data). A given subordinate being absent for some time because they are engaging in mutually exclusive activities (e.g., prospecting) is indeed one potential mechanism by which the observed sex difference in provisioning rate could

arise (i.e., a time allocation trade-off, which is captured under the rationale of the dispersal trade-off hypothesis). For a fuller treatment of this point please see our reply to the first point raised above (and our attention to this point in both the introduction [L90-92] and discussion [L423-427] of the manuscript).

L 527: what is a GdLMM? I am used to acronyms GLMM and GLM but not GdLMM

Authors' Response: Apologies. We only defined this acronym a little later in the original manuscript's text. We have now moved the definition up to the first usage of this term (L584; "generalised linear mixed models (GdLMMs)").

L 522: the full model approach can lead to ambiguity and retention of many terms that are not significant predictors of data, as well as the predictor:sample size ratio leading to uncertain conclusions of significance. Justify use of the this approach relative to these concerns.

Authors' Response: We took a full model approach because this is a conservative approach with respect to statistical significance (Burnham et al., 2011; Forstmeier & Schielzeth, 2011; Whittingham et al., 2006; see also Harrison et al., 2018) and it also provides model estimates for all variables of interest, which could then be used in the future in synthesis work and meta-analysis. We have now included a sentence to justify our approach in the Methods (L578-580).

Per comments above, the SM suggests that the provisioning rates were calculated from observations of the nest, in terms of next observation hours, not hours that the helpers was present (not prospecting) and available to help. This thus creates an issue: if a male is away 50% of the nest observation time, he will have a lower rate of provisioning per hour, even if he is not helping less. As in, in the hours he is there, he may be helping just as much. This could affect some of the conclusions made about this data.

Authors' Response: We have addressed this concern above (most extensively in the reply to the first comment made). We would be happy to clarify further if needed.

Tale S2: why was brood age categorical, not continuous? Brood age categorical, but brood size not? In Table S11, brood age seems continuous, whereas in other models it was not.

Authors' Response: Thank you for highlighting the lack of clarity here. We typically fitted brood age as a categorical variable to allow for the possibility of non-linear effects of variation in brood age (as it varies from 6 to 12 days in the data set). The one exception to this was in the model presented in Table S9 (previously Table S11) where (as explained in L679-681) the modest sample size gives us very low power to estimate model coefficients per brood age level. Nonetheless, we have checked that the main conclusions of the analysis presented in Table S9 (previously Table S11) are robust to including brood age as a categorical variable, and this is indeed the case: we still detect a significant negative effect of prospecting rate on provisioning rate (χ^2_1 = 4.87, p = 0.027). We have added this clarification in L680-681 of the Methods and have updated R scripts to include this additional model specification. We have also checked that our results in Table 2 (previously Table S2) are robust to including brood age as a continuous variable (sex effect: χ^2_1 = 16.76, p < 0.001).

References used in this response document:

Burnham, K. P., Anderson, D. R., & Huyvaert, K. P. (2011). AIC model selection and multimodel inference in behavioral ecology: Some background, observations, and comparisons. *Behavioral Ecology and Sociobiology*, *65*(1), 23–35. https://doi.org/10.1007/s00265-010-1029-6

Capilla-Lasheras, P., Harrison, X., Wood, E. M., Wilson, A. J., & Young, A. J. (2021). Altruistic bet hedging and the evolution of cooperation in a Kalahari bird. *Sci Adv*, *7*, 8980.

Downing, P. A., Griffin, A. S., & Cornwallis, C. K. (2018). Sex differences in helping effort reveal the effect of future reproduction on cooperative behaviour in birds. *Proceedings of the Royal Society B*, *285*, 20181164.

Forstmeier, W., & Schielzeth, H. (2011). Cryptic multiple hypotheses testing in linear models: Overestimated effect sizes and the winner's curse. *Behavioral Ecology and Sociobiology*, *65*(1), 47–55. https://doi.org/10.1007/s00265-010-1038-5

Harrison, X. A., Donaldson, L., Correa-Cano, M. E., Evans, J., Fisher, D. N., Goodwin, C. E. D., Robinson, B. S., Hodgson, D. J., & Inger, R. (2018). A brief introduction to mixed effects modelling and multi-model inference in ecology. *PeerJ*, *6*, e4794. https://doi.org/10.7717/peerj.4794

Walker, L., Tschirren, L., York, J., Sharp, P., Meddle, S., & Young, A. (2021). *Prolactin and the regulation of parental care and helping behavior in cooperatively breeding white-browed sparrow weaver societies*. bioRxiv

Webster, M. M., & Rutz, C. (2020). How STRANGE are your study animals? *Nature*, *582*(7812), 337–340. https://doi.org/10.1038/d41586-020-01751-5

Whittingham, M. J., Stephens, P. A., Bradbury, R. B., & Freckleton, R. P. (2006). Why do we still use stepwise modelling in ecology and behaviour? *Journal of Animal Ecology*, *75*(5), 1182–1189. https://doi.org/10.1111/j.1365-2656.2006.01141.x
